# Ultra-deep sequencing of HIV-1 near full-length and partial proviral genomes from recently infected blood donors at four blood centers in Brazil

**DOI:** 10.1186/1471-2334-14-S2-P58

**Published:** 2014-05-23

**Authors:** Rodrigo Pessôa, Jaqueline Tomoko Watanabe, Paula Loureiro, Maria Esther Lopes, Anna Barbara Carneiro-Proietti, Ester C Sabin, Michael P Busc, Sabri S Sanabani

**Affiliations:** 1 Institute of Tropical Medicine, University of São Paulo, São Paulo, Brazil; 2 Pernambuco Hematology and Hemotherapy Institute - HEMOPE, Recife, PE, Brazil; 3 Rio de Janeiro Hematology and Hemotherapy Institute - HEMORIO, Rio de Janeiro, Brazil; 4 Federal University of Minas Gerais - UFMG, Belo Horizonte, MG, Brazil; 5 Department of Infectious Diseases, University of São Paulo, São Paulo, Brazil; 6 Blood Systems Research Institute, San Francisco, California 94117, USA

## Background

Monitoring the genetic diversity of HIV-1 during the early stage of infection offers the best opportunity for understanding viral transmission dynamics and evolution of transmitted/founder viruses. Previous studies of HIV-infected donors in Brazil employing pol region sequencing have shown a predominance of subtype B (~80%) followed by F and C, with rare recombinant viruses. Here, we report application of high-throughput near-full-length (NFLG) and partial HIV-1 proviral genome deep sequencing to characterize HIV in recently infected blood donors at four major blood centers in Brazil.

## Methods

From 2007-2011, 341 HIV+ blood donors from 4 blood centers were recruited to participate in a case control study to identify risk exposure and motivation to donate. Forty-seven (17 from São Paulo [SP], 8 from Minas Gerais [MG], 11 from Pernambuco [PE] and 11 from Rio de Janeiro [RJ]) were classified as recently infected based on testing by less-sensitive (LS) or "detuned" enzyme immunoassay (Vironostika HIV-1 MicroElisa; bioMérieux, Durham, NC) or an LS chemiluminescent immunoassay (Vitros HIV-1/2 Assay; Ortho Diagnostics, Rochester, NY). Five overlapping amplicons spanning the HIV genome were PCR amplified from peripheral blood mononuclear cells (PBMCs). The amplicons were molecularly bar-coded, pooled, and sequenced by Illumina paired-end protocol.

## Results

Of the 47 recently infected donor samples studied, 39 (82.9%) NFLGs and 6 (12.7%) partial fragments were de novo assembled into contiguous sequences and successfully subtyped. Subtype B was the only non-recombinant virus characterized in this study and accounted for 60% (27/45) of samples. The remaining 40% (18/45) specimens showed various patterns of subtype discordance in different regions of HIV-1 genomes indicating 2-4 circulating recombinant subtypes derived from clades B, F and C. Over 50% of infection in MG and RJ harbor unique inter-subtype recombinant forms.

## Conclusion

Our findings revealed a high proportion of HIV-1 recombinants among recently infected blood donors in Brazil which has implications for future diagnosis, therapy and efficient vaccine development.

